# A [Cu_3_(μ_3_-O)]–pyrazolate metallacycle with terminal nitrate ligands exhibiting point group symmetry 3

**DOI:** 10.1107/S2056989016003741

**Published:** 2016-03-15

**Authors:** Logesh Mathivathanan, Raquel Cruz, Raphael G. Raptis

**Affiliations:** a Department of Chemistry, University of Puerto Rico – Rio Piedras, San Juan 00936, Puerto Rico, USA

**Keywords:** crystal structure, copper–pyrazolate complex, terminal nitrate ligands

## Abstract

The trinuclear triangular cuprate anion of the title compound, tris­[bis­(tri­phenyl­phospho­ranyl­idene)ammonium] tris­(μ_2_-4-chloro­pyrazolato-κ^2^ *N*:*N*′)-μ_3_-oxido-tris­[(nitrato-κ^2^ *O*,*O*′)cuprate(II)] nitrate monohydrate, (C_36_H_30_P_2_N)[Cu_3_(C_3_H_2_ClN_2_)_3_(NO_3_)_3_O]NO_3_·H_2_O, has point group symmetry 3., with the μ_3_-O atom located on the threefold rotation axis. The distorted square-pyramidal coordination sphere of the Cu^II^ atom is completed by two N atoms of *trans*-bridging pyrazolate groups and a chelating nitrate anion. The complex anion is slightly bent, with the nitrate and pyrazolate groups occupying positions above and below the Cu_3_ plane, respectively. In the crystal, weak O—H⋯O and C—H⋯O hydrogen bonds, as well as π–π inter­actions, are present.

## Abstract 2

The cuprate anion in the title compound is located about a threefold rotation axis and hence forms an almost planar Cu_3_(μ_3_-O)-core, where the μ_3_-O atom is located 0.12 Å above the Cu_3_ plane.

## Chemical context   

Trinuclear copper complexes with a triangular arrangement of the copper(II) cations are of importance in terms of their magnetic and redox properties (Rivera-Carrillo *et al.*, 2008 ▸). Moreover, Cu_3_(μ_3_-O/OH) moieties make up the active sites of several multicopper oxidase enzymes (Solomon *et al.*, 2014 ▸). Pyrazolate anions as ligands are of bidentate chelating nature and are able to bind to the the Cu^II^ cations in suitable angles to form triangular complexes (Halcrow, 2009 ▸; Viciano-Chumillas *et al.*, 2010 ▸).
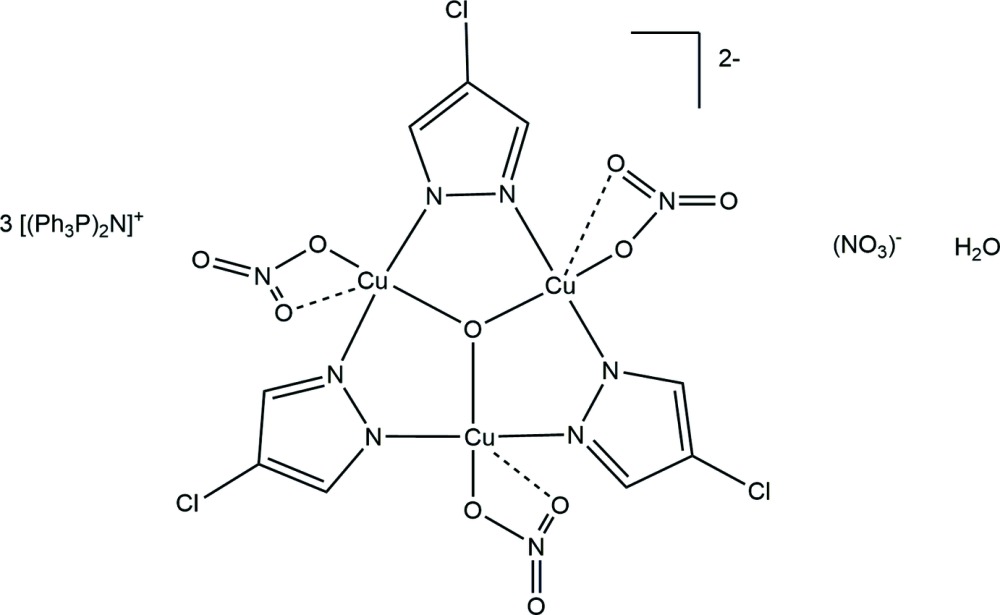


Nitrato and pyrazolato ligands are commonly studied ligands in Cu^II^ coordination chemistry. Simple Cu^II^ nitrate complexes are aplenty in the literature and have been studied in detail with respect to their part in the nitro­gen cycle. Triangular trinuclear Cu^II^ complexes with terminal nitrate ligands, however, are scarcer (Alsalme *et al.*, 2014 ▸). Nitrates, being good hydrogen-bonding acceptors, are able to form Cu_3_(μ_3_-OH) complexes, with hydrogen bonds to the μ_3_-OH group and to ancilliary ligands and water mol­ecules.

In this communication we describe the accidental synthesis and the structure of a trinuclear Cu–pyrazolato complex, *viz*. (PPN)_3_[Cu_3_(μ_3_-O)(μ-4-Clpz)_3_(NO_3_)_3_](NO_3_)·H_2_O, where PPN = bis­(tri­phenyl­phospho­ranyl­idene)ammonium; 4-Cl-pz = 4-chloropyrazolate. A related Cu_3_-pyrazolato complex was reported by Angaridis *et al.* (2002 ▸).

## Structural commentary   

**Figure 1:**
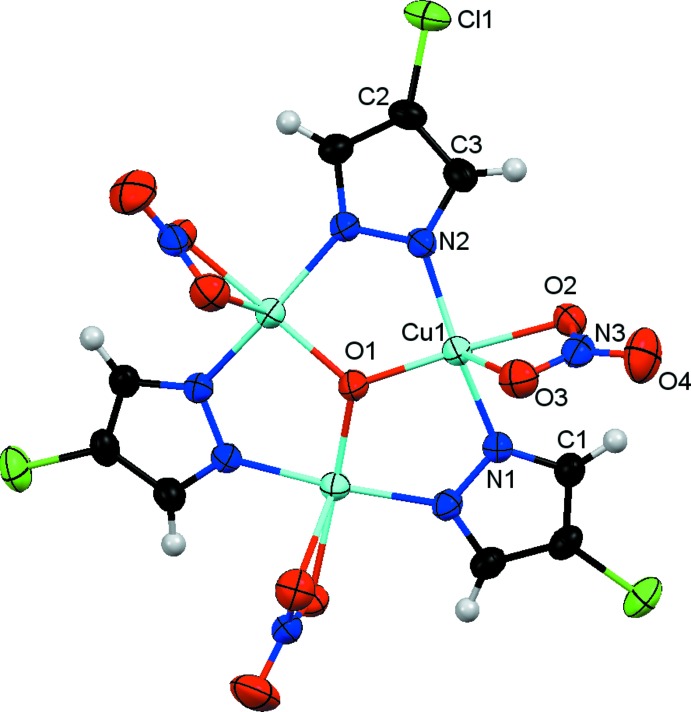
The mol­ecular structure of the trinuclear pyrazolatocuprate anion in the title compound showing the atom-labeling scheme for the symmetry-independent atoms. Non-H atoms are shown as displacement ellipsoids at the 30% probability level.

The nine-membered metallacycle Cu_3_N_6_ in the cuprate anion (Fig. 1 ▸) is strung together by a μ_3_-O group located at the center of the triangle (point group symmetry of the complete mol­ecule 3.), forming an almost planar Cu_3_(μ_3_-O)-core, where the μ_3_-O atom O1 is located 0.122 (7) Å above the Cu_3_ plane. The distorted square-pyramidal geometry of the Cu^II^ atom is completed by the two N atoms of symmetry-related *trans-*bridging pyrazolato ligands, and a terminal nitrato ligand that is bound to the metal in a chelating fashion (Table 1 ▸). The complex is slightly bent with the nitrate and pyrazolato groups occupying positions above and below the Cu_3_ plane, respectively. The Cl atom of the pyrazole anion is located approximately 1.28 Å below the Cu_3_ plane. The non-coordinating nitrate counter-anion is located about a special position with the nitro­gen atom on the threefold rotation axis.

The tri­phenyl­phosphene groups in the PPN cation are staggered around the central N atom [P—N—P angle 139.5 (2)°] and show bond lengths and angles characteristic for this unit (Beckett *et al.*, 2010 ▸).

## Supra­molecular features   

**Figure 2:**
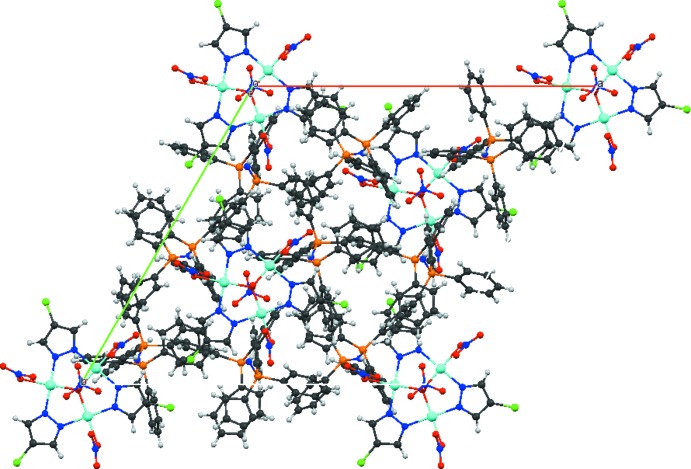
The crystal packing diagram for the title compound shown down [001].

The interstitial water O atom is also located on a threefold rotation axis which consequently results in disordered H atoms of this moiety. Although these H atoms could not be located, three O⋯O distances to the chelating nitrate anions of 3.367 (6) Å point to weak O—H⋯O hydrogen bonds in the structure. This nitrate O atom is additionally involved in weak non-classical hydrogen-bonding inter­actions with one of the C–H groups of the PPN cation (Table 2 ▸). The latter shows also π–π inter­actions [3.902 (7) Å] with one of the pyrazolate rings, leading to an overall three-dimensional network. The packing of the mol­ecular units is shown in Fig. 2 ▸.

## Synthesis and crystallization   

4-Cl-pz and the hexa­nuclear Cu_6_-pyrazolato complex (PPN)[{Cu_3_(μ_3_-O)(μ-4-Cl-pz)_3_}_2_(μ-3,5-Ph_2_pz)_3_], were synthesized by published procedures (Maresca *et al.*, 1997 ▸; Mezei *et al.*, 2007 ▸). (NH_4_)_2_Ce(NO_3_)_6_ (2 eq.) was dissolved in 5 ml of aceto­nitrile and was layered over a CH_2_Cl_2_ solution of the hexa­nuclear copper(II) complex (1 eq.). Slow mixing of the reactants and solvent evaporation over a few weeks yielded dark-blue crystals of the title compound.

## Refinement   

Crystal data, data collection and structure refinement details are summarized in Table 3 ▸. The C-bound H atoms were placed geometrically, with C—H = 0.93 Å and *U*_iso_(H) = 1.2*U*_eq_(C). The isolated water solvent O atom, O1*W*, was refined isotropically. H atoms bound to the water oxygen atom could not be placed satisfactorily with agreeable occupancy as O1*W* resides on a threefold rotation axis, resulting in crystallographically disordered H atoms. These H atoms were not modelled but are included in the formula of the title compound.

## Supplementary Material

Crystal structure: contains datablock(s) I. DOI: 10.1107/S2056989016003741/wm4004sup1.cif

Supporting information file. DOI: 10.1107/S2056989016003741/wm4004Isup3.cdx

Structure factors: contains datablock(s) I. DOI: 10.1107/S2056989016003741/wm4004Isup4.hkl

CCDC reference: 1458061

Additional supporting information:  crystallographic information; 3D view; checkCIF report

## Figures and Tables

**Table 1:** Selected bond lengths (Å)

Cu1—O1	1.8816 (7)	Cu1—O2	2.059 (3)
Cu1—N2	1.952 (4)	Cu1—O3	2.483 (4)
Cu1—N1	1.960 (4)		

**Table 2:** Hydrogen-bond geometry (Å, °)

*D*—H⋯*A*	*D*—H	H⋯*A*	*D*⋯*A*	*D*—H⋯*A*
C13—H13⋯O3^i^	0.93	2.53	3.410 (11)	157

Symmetry code: (i) 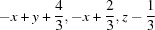.

**Table 3:** Experimental details

Crystal data	
Chemical formula	(C_36_H_30_P_2_N)[Cu_3_(C_3_H_2_ClN_2_)_3_(NO_3_)_3_O]NO_3_·H_2_O
*M*_r_	2390.85
Crystal system, space group	Trigonal, *R*3
Temperature (K)	296
*a*, *c* (Å)	23.038 (2), 18.4214 (17)
*V* (Å^3^)	8466.9 (17)
*Z*	3
Radiation type	Mo *K*α
μ (mm^−1^)	0.79
Crystal size (mm)	0.23 × 0.14 × 0.13

Data collection	
Diffractometer	Bruker APEXII CCD
Absorption correction	Multi-scan (*SADABS*; Bruker, 2005 ▸)
*T*_min_, *T*_max_	0.840, 0.905
No. of measured, independent and observed [*I* > 2σ(*I*)] reflections	30414, 7675, 6428
*R*_int_	0.033
(sin θ/λ)_max_ (Å^−1^)	0.625

Refinement	
*R*[*F* ^2^ > 2σ(*F* ^2^)], *wR*(*F* ^2^), *S*	0.034, 0.088, 1.03
No. of reflections	7675
No. of parameters	468
No. of restraints	1
H-atom treatment	H-atom parameters constrained
Δρ_max_, Δρ_min_ (e Å^−3^)	0.41, −0.29
Absolute structure	Flack *x* determined using 2750 quotients [(*I* ^+^)−(*I* ^−^)]/[(*I* ^+^)+(*I* ^−^)] (Parsons *et al.*, 2013 ▸)
Absolute structure parameter	0.004 (5)

Computer programs: *APEX2* and *SAINT* (Bruker, 2005 ▸), *SHELXS97* (Sheldrick, 2008 ▸), *SHELXL2014* (Sheldrick, 2015 ▸), *Mercury* (Macrae *et al.*, 2006 ▸) and *publCIF* (Westrip, 2010 ▸).

## supplementary crystallographic information

### Crystal data

**Table:**

(C_36_H_30_P_2_N)[Cu_3_(C_3_H_2_ClN_2_)_3_(NO_3_)_3_O]NO_3_·H_2_O	*D*_x_ = 1.407 Mg m^−^^3^
*M**_r_* = 2390.85	Mo *K*α radiation, λ = 0.71073 Å
Trigonal, *R*3	Cell parameters from 9882 reflections
*a* = 23.038 (2) Å	θ = 2.3–26.0°
*c* = 18.4214 (17) Å	µ = 0.79 mm^−^^1^
*V* = 8466.9 (17) Å^3^	*T* = 296 K
*Z* = 3	Polygon, blue
*F*(000) = 3687	0.23 × 0.14 × 0.13 mm

### Data collection

**Table:**

Bruker APEXII CCD diffractometer	7675 independent reflections
Radiation source: sealed tube	6428 reflections with *I* > 2σ(*I*)
Graphite monochromator	*R*_int_ = 0.033
φ and ω scans	θ_max_ = 26.4°, θ_min_ = 2.3°
Absorption correction: multi-scan (*SADABS*; Bruker, 2005)	*h* = −28→28
*T*_min_ = 0.840, *T*_max_ = 0.905	*k* = −28→28
30414 measured reflections	*l* = −22→22

### Refinement

**Table:**

Refinement on *F*^2^	Hydrogen site location: inferred from neighbouring sites
Least-squares matrix: full	H-atom parameters constrained
*R*[*F*^2^ > 2σ(*F*^2^)] = 0.034	*w* = 1/[σ^2^(*F*_o_^2^) + (0.0468*P*)^2^ + 0.1352*P*] where *P* = (*F*_o_^2^ + 2*F*_c_^2^)/3
*wR*(*F*^2^) = 0.088	(Δ/σ)_max_ = 0.001
*S* = 1.03	Δρ_max_ = 0.41 e Å^−^^3^
7675 reflections	Δρ_min_ = −0.29 e Å^−^^3^
468 parameters	Absolute structure: Flack *x* determined using 2750 quotients [(*I*^+^)-(*I*^-^)]/[(*I*^+^)+(*I*^-^)] (Parsons *et al.*, 2013)
1 restraint	Absolute structure parameter: 0.004 (5)

### Special details

**Table:**

Geometry. All e.s.d.'s (except the e.s.d. in the dihedral angle between two l.s. planes) are estimated using the full covariance matrix. The cell e.s.d.'s are taken into account individually in the estimation of e.s.d.'s in distances, angles and torsion angles; correlations between e.s.d.'s in cell parameters are only used when they are defined by crystal symmetry. An approximate (isotropic) treatment of cell e.s.d.'s is used for estimating e.s.d.'s involving l.s. planes.

### Fractional atomic coordinates and isotropic or equivalent isotropic displacement parameters (Å^2^)

**Table:**

	*x*	*y*	*z*	*U*_iso_*/*U*_eq_	
O1W	0.0000	1.0000	0.3698 (7)	0.141 (4)*	
Cu1	0.57819 (3)	0.31688 (3)	0.88140 (3)	0.04893 (15)	
O1	0.6667	0.3333	0.8748 (4)	0.0691 (18)	
O2	0.48058 (17)	0.29648 (18)	0.8736 (2)	0.0619 (9)	
N1	0.54504 (18)	0.22155 (19)	0.8997 (2)	0.0504 (9)	
N2	0.61014 (18)	0.41208 (18)	0.8960 (2)	0.0493 (9)	
N3	0.4670 (2)	0.28251 (19)	0.8087 (3)	0.0582 (10)	
C1	0.4851 (2)	0.1710 (2)	0.9186 (3)	0.0590 (12)	
H1	0.4465	0.1738	0.9249	0.071*	
C2	0.4893 (3)	0.1144 (2)	0.9272 (3)	0.0570 (12)	
C3	0.5783 (3)	0.4455 (3)	0.9126 (3)	0.0582 (12)	
H3	0.5321	0.4274	0.9141	0.070*	
Cl1	0.42537 (8)	0.03570 (7)	0.95132 (10)	0.0859 (5)	
O3	0.5141 (2)	0.2937 (2)	0.7664 (2)	0.0753 (10)	
O4	0.4095 (2)	0.2572 (3)	0.7874 (3)	0.1105 (17)	
P1	0.81315 (5)	0.23893 (5)	0.23155 (5)	0.0379 (2)	
P2	0.78221 (5)	0.16748 (5)	0.37198 (5)	0.0392 (2)	
N4	0.78666 (18)	0.21790 (18)	0.31150 (18)	0.0461 (8)	
C4	0.7627 (2)	0.1937 (2)	0.4558 (2)	0.0440 (10)	
C5	0.7353 (2)	0.2344 (2)	0.4544 (2)	0.0487 (11)	
H5	0.7293	0.2500	0.4101	0.058*	
C6	0.7165 (3)	0.2526 (3)	0.5183 (3)	0.0625 (13)	
H6	0.6978	0.2803	0.5167	0.075*	
C7	0.7254 (3)	0.2299 (3)	0.5829 (3)	0.0754 (17)	
H7	0.7129	0.2424	0.6256	0.090*	
C8	0.7527 (3)	0.1887 (3)	0.5860 (3)	0.0788 (17)	
H8	0.7584	0.1732	0.6305	0.095*	
C9	0.7717 (3)	0.1703 (3)	0.5224 (3)	0.0615 (13)	
H9	0.7904	0.1427	0.5242	0.074*	
C10	0.8593 (2)	0.1669 (2)	0.3837 (2)	0.0463 (10)	
C11	0.9135 (2)	0.2251 (3)	0.4090 (3)	0.0610 (12)	
H11	0.9081	0.2606	0.4241	0.073*	
C12	0.9761 (3)	0.2304 (4)	0.4120 (3)	0.0801 (18)	
H12	1.0128	0.2696	0.4289	0.096*	
C13	0.9836 (3)	0.1783 (4)	0.3899 (3)	0.0796 (18)	
H13	1.0257	0.1819	0.3922	0.095*	
C14	0.9302 (3)	0.1205 (4)	0.3645 (4)	0.0833 (18)	
H14	0.9363	0.0855	0.3490	0.100*	
C15	0.8679 (3)	0.1139 (3)	0.3617 (3)	0.0629 (13)	
H15	0.8315	0.0743	0.3451	0.075*	
C16	0.7162 (2)	0.0829 (2)	0.3546 (2)	0.0467 (10)	
C17	0.6790 (2)	0.0681 (3)	0.2917 (3)	0.0569 (12)	
H17	0.6875	0.1021	0.2589	0.068*	
C18	0.6291 (3)	0.0032 (3)	0.2771 (3)	0.0732 (15)	
H18	0.6053	−0.0064	0.2338	0.088*	
C19	0.6146 (3)	−0.0466 (3)	0.3259 (4)	0.0771 (17)	
H19	0.5810	−0.0902	0.3158	0.093*	
C20	0.6493 (3)	−0.0327 (3)	0.3896 (4)	0.0807 (18)	
H20	0.6381	−0.0665	0.4236	0.097*	
C21	0.7012 (3)	0.0315 (3)	0.4039 (3)	0.0737 (16)	
H21	0.7260	0.0403	0.4465	0.088*	
C22	0.8529 (2)	0.1956 (2)	0.1928 (2)	0.0414 (9)	
C23	0.8160 (2)	0.1331 (2)	0.1628 (3)	0.0532 (11)	
H23	0.7698	0.1141	0.1578	0.064*	
C24	0.8467 (3)	0.0979 (3)	0.1398 (3)	0.0678 (14)	
H24	0.8214	0.0559	0.1185	0.081*	
C25	0.9149 (3)	0.1251 (3)	0.1483 (3)	0.0666 (14)	
H25	0.9355	0.1010	0.1339	0.080*	
C26	0.9520 (3)	0.1870 (3)	0.1778 (3)	0.0630 (13)	
H26	0.9980	0.2055	0.1828	0.076*	
C27	0.9219 (2)	0.2228 (2)	0.2003 (3)	0.0505 (10)	
H27	0.9477	0.2652	0.2204	0.061*	
C28	0.7444 (2)	0.2268 (2)	0.1749 (2)	0.0415 (9)	
C29	0.6909 (2)	0.2287 (2)	0.2075 (3)	0.0526 (11)	
H29	0.6902	0.2334	0.2575	0.063*	
C30	0.6394 (2)	0.2237 (3)	0.1659 (3)	0.0674 (14)	
H30	0.6037	0.2250	0.1880	0.081*	
C31	0.6397 (3)	0.2167 (3)	0.0916 (3)	0.0738 (16)	
H31	0.6048	0.2140	0.0636	0.089*	
C32	0.6910 (3)	0.2138 (3)	0.0599 (3)	0.0698 (15)	
H32	0.6906	0.2081	0.0098	0.084*	
C33	0.7439 (3)	0.2192 (3)	0.1000 (2)	0.0578 (12)	
H33	0.7791	0.2176	0.0772	0.069*	
C34	0.8736 (2)	0.3267 (2)	0.2287 (3)	0.0468 (10)	
C35	0.8897 (3)	0.3652 (3)	0.2894 (4)	0.0845 (19)	
H35	0.8686	0.3462	0.3331	0.101*	
C36	0.9372 (5)	0.4322 (3)	0.2866 (5)	0.123 (3)	
H36	0.9495	0.4580	0.3287	0.147*	
C37	0.9664 (4)	0.4611 (3)	0.2211 (5)	0.106 (3)	
H37	0.9980	0.5065	0.2190	0.127*	
C38	0.9497 (3)	0.4245 (3)	0.1612 (4)	0.0813 (18)	
H38	0.9697	0.4445	0.1173	0.098*	
C39	0.9028 (3)	0.3568 (2)	0.1633 (3)	0.0632 (13)	
H39	0.8908	0.3316	0.1208	0.076*	
N5	0.6667	0.3333	0.3566 (5)	0.069 (2)	
O5	0.6275 (3)	0.2721 (2)	0.3545 (3)	0.1083 (16)	

### Atomic displacement parameters (Å^2^)

**Table:**

	*U*^11^	*U*^22^	*U*^33^	*U*^12^	*U*^13^	*U*^23^
Cu1	0.0436 (3)	0.0458 (3)	0.0582 (3)	0.0230 (3)	0.0029 (3)	0.0017 (3)
O1	0.044 (2)	0.044 (2)	0.119 (6)	0.0220 (10)	0.000	0.000
O2	0.058 (2)	0.069 (2)	0.065 (2)	0.0356 (18)	0.0048 (17)	−0.0040 (18)
N1	0.044 (2)	0.046 (2)	0.060 (2)	0.0217 (18)	0.0012 (17)	−0.0010 (17)
N2	0.049 (2)	0.048 (2)	0.054 (2)	0.0268 (19)	0.0061 (17)	0.0045 (17)
N3	0.054 (3)	0.048 (2)	0.073 (3)	0.025 (2)	−0.011 (2)	0.007 (2)
C1	0.047 (3)	0.053 (3)	0.069 (3)	0.019 (2)	0.005 (2)	−0.002 (2)
C2	0.059 (3)	0.045 (3)	0.051 (3)	0.014 (2)	−0.001 (2)	0.005 (2)
C3	0.060 (3)	0.061 (3)	0.062 (3)	0.037 (3)	0.011 (2)	0.014 (2)
Cl1	0.0748 (10)	0.0513 (8)	0.0996 (11)	0.0075 (7)	0.0071 (8)	0.0112 (7)
O3	0.084 (3)	0.078 (3)	0.064 (2)	0.040 (2)	0.008 (2)	0.0030 (19)
O4	0.068 (3)	0.129 (4)	0.118 (4)	0.037 (3)	−0.030 (3)	0.004 (3)
P1	0.0386 (6)	0.0401 (6)	0.0386 (5)	0.0224 (5)	0.0019 (4)	0.0017 (4)
P2	0.0378 (5)	0.0423 (6)	0.0376 (5)	0.0200 (5)	−0.0016 (4)	0.0010 (4)
N4	0.053 (2)	0.055 (2)	0.0393 (18)	0.0341 (19)	0.0026 (16)	0.0058 (16)
C4	0.038 (2)	0.044 (2)	0.040 (2)	0.0136 (19)	−0.0028 (17)	−0.0008 (18)
C5	0.046 (2)	0.050 (3)	0.046 (3)	0.021 (2)	0.0003 (19)	−0.0051 (19)
C6	0.055 (3)	0.063 (3)	0.064 (3)	0.025 (3)	0.010 (2)	−0.008 (3)
C7	0.068 (4)	0.094 (4)	0.054 (3)	0.033 (3)	0.009 (3)	−0.020 (3)
C8	0.083 (4)	0.100 (5)	0.036 (3)	0.033 (4)	0.001 (2)	0.007 (3)
C9	0.071 (3)	0.076 (3)	0.043 (3)	0.041 (3)	−0.003 (2)	0.003 (2)
C10	0.041 (2)	0.057 (3)	0.044 (2)	0.027 (2)	0.0013 (18)	0.008 (2)
C11	0.048 (3)	0.065 (3)	0.066 (3)	0.026 (3)	−0.007 (2)	−0.005 (3)
C12	0.042 (3)	0.103 (5)	0.078 (4)	0.023 (3)	−0.008 (3)	0.005 (3)
C13	0.057 (4)	0.120 (6)	0.077 (4)	0.056 (4)	0.006 (3)	0.027 (4)
C14	0.081 (4)	0.098 (5)	0.099 (5)	0.066 (4)	0.006 (4)	0.009 (4)
C15	0.059 (3)	0.061 (3)	0.080 (3)	0.038 (3)	0.004 (3)	0.008 (3)
C16	0.040 (2)	0.046 (2)	0.054 (3)	0.022 (2)	0.0006 (19)	0.000 (2)
C17	0.048 (3)	0.059 (3)	0.056 (3)	0.022 (2)	−0.009 (2)	−0.003 (2)
C18	0.055 (3)	0.079 (4)	0.071 (3)	0.023 (3)	−0.013 (3)	−0.020 (3)
C19	0.051 (3)	0.055 (3)	0.105 (5)	0.011 (3)	−0.001 (3)	−0.022 (3)
C20	0.073 (4)	0.048 (3)	0.103 (5)	0.017 (3)	−0.004 (3)	0.009 (3)
C21	0.069 (3)	0.055 (3)	0.077 (4)	0.016 (3)	−0.015 (3)	0.011 (3)
C22	0.041 (2)	0.046 (2)	0.042 (2)	0.026 (2)	0.0018 (17)	0.0011 (18)
C23	0.044 (2)	0.046 (3)	0.067 (3)	0.021 (2)	0.002 (2)	−0.006 (2)
C24	0.067 (3)	0.050 (3)	0.090 (4)	0.032 (3)	0.005 (3)	−0.016 (3)
C25	0.069 (4)	0.063 (3)	0.087 (4)	0.047 (3)	0.020 (3)	0.005 (3)
C26	0.045 (3)	0.068 (3)	0.085 (4)	0.035 (3)	0.012 (2)	0.008 (3)
C27	0.042 (2)	0.047 (3)	0.065 (3)	0.025 (2)	0.002 (2)	−0.001 (2)
C28	0.044 (2)	0.042 (2)	0.043 (2)	0.0243 (19)	0.0007 (17)	0.0019 (17)
C29	0.053 (3)	0.062 (3)	0.052 (3)	0.036 (2)	0.002 (2)	0.000 (2)
C30	0.049 (3)	0.077 (4)	0.086 (4)	0.038 (3)	−0.006 (3)	−0.004 (3)
C31	0.069 (4)	0.079 (4)	0.082 (4)	0.044 (3)	−0.026 (3)	−0.001 (3)
C32	0.080 (4)	0.088 (4)	0.049 (3)	0.047 (3)	−0.013 (3)	0.004 (3)
C33	0.064 (3)	0.076 (3)	0.045 (3)	0.043 (3)	0.001 (2)	0.006 (2)
C34	0.046 (2)	0.038 (2)	0.059 (3)	0.023 (2)	0.004 (2)	−0.0018 (19)
C35	0.089 (4)	0.055 (3)	0.077 (4)	0.011 (3)	0.013 (3)	−0.009 (3)
C36	0.157 (8)	0.054 (4)	0.106 (6)	0.015 (4)	0.015 (5)	−0.025 (4)
C37	0.084 (5)	0.045 (3)	0.166 (8)	0.015 (3)	0.029 (5)	0.000 (4)
C38	0.074 (4)	0.055 (3)	0.118 (5)	0.035 (3)	0.035 (4)	0.021 (4)
C39	0.064 (3)	0.049 (3)	0.074 (3)	0.026 (3)	0.017 (3)	0.009 (2)
N5	0.065 (3)	0.065 (3)	0.077 (5)	0.0327 (16)	0.000	0.000
O5	0.090 (3)	0.074 (3)	0.150 (5)	0.032 (3)	0.020 (3)	0.000 (3)

### Geometric parameters (Å, º)

**Table:**

Cu1—O1	1.8816 (7)	C10—C15	1.393 (7)
Cu1—N2	1.952 (4)	C11—C12	1.385 (8)
Cu1—N1	1.960 (4)	C12—C13	1.360 (9)
Cu1—O2	2.059 (3)	C13—C14	1.366 (9)
Cu1—O3	2.483 (4)	C14—C15	1.367 (8)
O1—Cu1^i^	1.8816 (7)	C16—C17	1.379 (6)
O1—Cu1^ii^	1.8816 (7)	C16—C21	1.391 (7)
O2—N3	1.237 (5)	C17—C18	1.382 (7)
N1—C1	1.334 (6)	C18—C19	1.362 (9)
N1—N2^i^	1.345 (5)	C19—C20	1.365 (9)
N2—C3	1.337 (6)	C20—C21	1.386 (8)
N2—N1^ii^	1.345 (5)	C22—C23	1.371 (6)
N3—O4	1.215 (6)	C22—C27	1.393 (6)
N3—O3	1.253 (6)	C23—C24	1.382 (7)
C1—C2	1.364 (7)	C24—C25	1.379 (8)
C2—C3^i^	1.368 (7)	C25—C26	1.356 (8)
C2—Cl1	1.727 (5)	C26—C27	1.381 (6)
C3—C2^ii^	1.368 (7)	C28—C33	1.389 (6)
P1—N4	1.575 (4)	C28—C29	1.391 (6)
P1—C34	1.794 (4)	C29—C30	1.368 (7)
P1—C28	1.799 (4)	C30—C31	1.379 (8)
P1—C22	1.805 (4)	C31—C32	1.350 (8)
P2—N4	1.576 (4)	C32—C33	1.378 (7)
P2—C10	1.795 (4)	C34—C35	1.358 (7)
P2—C4	1.795 (4)	C34—C39	1.386 (7)
P2—C16	1.802 (4)	C35—C36	1.376 (9)
C4—C5	1.366 (6)	C36—C37	1.379 (11)
C4—C9	1.397 (6)	C37—C38	1.323 (10)
C5—C6	1.391 (7)	C38—C39	1.384 (8)
C6—C7	1.356 (8)	N5—O5^i^	1.238 (5)
C7—C8	1.379 (9)	N5—O5	1.238 (5)
C8—C9	1.388 (8)	N5—O5^ii^	1.238 (5)
C10—C11	1.378 (7)		
			
O1—Cu1—N2	91.18 (12)	C8—C9—C4	119.5 (5)
O1—Cu1—N1	90.73 (12)	C11—C10—C15	119.6 (4)
N2—Cu1—N1	162.20 (16)	C11—C10—P2	117.0 (4)
O1—Cu1—O2	172.2 (2)	C15—C10—P2	123.2 (4)
N2—Cu1—O2	91.22 (15)	C10—C11—C12	119.8 (5)
N1—Cu1—O2	89.25 (14)	C13—C12—C11	119.8 (6)
Cu1—O1—Cu1^i^	119.59 (5)	C12—C13—C14	120.8 (5)
Cu1—O1—Cu1^ii^	119.59 (5)	C13—C14—C15	120.4 (6)
Cu1^i^—O1—Cu1^ii^	119.59 (5)	C14—C15—C10	119.6 (6)
N3—O2—Cu1	103.4 (3)	C17—C16—C21	118.6 (4)
C1—N1—N2^i^	108.1 (4)	C17—C16—P2	120.0 (4)
C1—N1—Cu1	132.6 (3)	C21—C16—P2	121.4 (4)
N2^i^—N1—Cu1	119.3 (3)	C16—C17—C18	120.6 (5)
C3—N2—N1^ii^	108.3 (4)	C19—C18—C17	120.3 (5)
C3—N2—Cu1	132.1 (3)	C18—C19—C20	120.1 (5)
N1^ii^—N2—Cu1	118.9 (3)	C19—C20—C21	120.4 (6)
O4—N3—O2	120.7 (5)	C20—C21—C16	119.9 (5)
O4—N3—O3	121.5 (5)	C23—C22—C27	118.8 (4)
O2—N3—O3	117.9 (4)	C23—C22—P1	121.4 (3)
N1—C1—C2	109.1 (4)	C27—C22—P1	119.4 (3)
C1—C2—C3^i^	105.8 (4)	C22—C23—C24	120.6 (4)
C1—C2—Cl1	127.0 (4)	C25—C24—C23	120.0 (5)
C3^i^—C2—Cl1	127.2 (4)	C26—C25—C24	119.9 (5)
N2—C3—C2^ii^	108.6 (4)	C25—C26—C27	120.6 (5)
N4—P1—C34	109.7 (2)	C26—C27—C22	120.1 (5)
N4—P1—C28	108.61 (19)	C33—C28—C29	118.8 (4)
C34—P1—C28	106.6 (2)	C33—C28—P1	123.1 (3)
N4—P1—C22	115.14 (19)	C29—C28—P1	118.1 (3)
C34—P1—C22	106.8 (2)	C30—C29—C28	120.1 (5)
C28—P1—C22	109.62 (19)	C29—C30—C31	120.6 (5)
N4—P2—C10	113.0 (2)	C32—C31—C30	119.5 (5)
N4—P2—C4	107.2 (2)	C31—C32—C33	121.3 (5)
C10—P2—C4	108.3 (2)	C32—C33—C28	119.7 (5)
N4—P2—C16	112.4 (2)	C35—C34—C39	118.9 (5)
C10—P2—C16	108.4 (2)	C35—C34—P1	121.2 (4)
C4—P2—C16	107.4 (2)	C39—C34—P1	119.9 (4)
P1—N4—P2	139.5 (2)	C34—C35—C36	120.4 (6)
C5—C4—C9	119.3 (4)	C35—C36—C37	119.7 (7)
C5—C4—P2	119.6 (3)	C38—C37—C36	120.5 (6)
C9—C4—P2	121.0 (4)	C37—C38—C39	120.6 (6)
C4—C5—C6	120.8 (5)	C38—C39—C34	119.8 (5)
C7—C6—C5	119.7 (5)	O5^i^—N5—O5	119.91 (5)
C6—C7—C8	120.7 (5)	O5^i^—N5—O5^ii^	119.90 (6)
C7—C8—C9	119.8 (5)	O5—N5—O5^ii^	119.91 (5)

Symmetry codes: (i) −*y*+1, *x*−*y*, *z*; (ii) −*x*+*y*+1, −*x*+1, *z*.

### Hydrogen-bond geometry (Å, º)

**Table:**

*D*—H···*A*	*D*—H	H···*A*	*D*···*A*	*D*—H···*A*
C13—H13···O3^iii^	0.93	2.53	3.410 (11)	157

Symmetry code: (iii) −*x*+*y*+4/3, −*x*+2/3, *z*−1/3.
